# “I Need to Get My Culture Back”: Youth and Provider Perspectives on Integrating Culturally Based Approaches into Sexual and Reproductive Health Programs for Native Hawaiian and Pacific Islander Youth Experiencing Homelessness

**DOI:** 10.1007/s11121-023-01573-7

**Published:** 2023-08-11

**Authors:** Charlene Kuo, Michelle Jasczynski, Jee Hun Yoo, Jennifer L. Robinson, Katelyn Reynolds, Lisa Anoruo, Kayla Bae, Lana Sue Ka‘opua, Rebecca Chavez, Jacqueline Tellei, Elizabeth M. Aparicio

**Affiliations:** 1 School of Public Health, Department of Behavioral and Community Health, University of Maryland, College Park, MD USA; 2 College of Computer, Mathematical, & Natural Sciences, Department of Biology, University of Maryland, College Park, MD USA; 3 School of Public Health, Department of Public Health Science, University of Maryland, College Park, MD USA; 4 Thompson School of Social Work & Public Health (Retired), Department of Social Work, University of Hawai‘i at Mānoa, Honolulu, HI USA; 5 Waikiki Health, Honolulu, HI USA; 6 PATH Clinic and Youth Outreach, Honolulu, HI USA

**Keywords:** Sexual and reproductive health, Native Hawaiian, Pacific Islander, Qualitative, Culturally based approaches, Culture

## Abstract

There is growing interest in decolonizing sexual and reproductive health (SRH) and embedding cultural practices into social and medical services in Hawai‘i. Wahine (“woman”) Talk is a multilevel, comprehensive SRH program for female youth experiencing homelessness (YEH) led by community health, social work, and medical providers. This study examines youth and program provider perspectives of culturally based approaches that may strengthen SRH programs. The study team conducted three focus groups and ten in-depth interviews with participating youth and program providers after the program’s conclusion. Youth participants were aged 14 to 22 years (*M* = 18.1) and of Native Hawaiian or Pacific Islander ancestry. Interview transcripts were analyzed using structured thematic analysis. The youth described feeling estranged from their ancestral cultures and suggested incorporating multiple cultural practices to enhance their connection to community, body, and land into SRH programming for YEH. They identified several *ʻāina* (“land”)–based approaches, hands-on learning, *hula*, and language as possible practices to weave into the program. While youth felt estranged from their ancestral cultures, they discussed Native Hawaiian and Pacific Islander health perspectives where *ʻāina* and relationships are considered life-sustaining. Youth and program staff stressed incorporating culture respectfully, caring for the whole person, and providing trauma-informed care. Future policy, practice, and research should consider protecting and integrating Native Hawaiian conceptions of health into SRH policy and practice and include youths’ cultural identities in SRH intervention development.

## Introduction

Researchers have linked multiple distal social determinants (i.e., Western colonization, historical trauma, cultural dispossession, land dispossession, and marginalization) to present-day health disparities experienced by Indigenous peoples (Goodyear-Kaʻōpua, 2013; Ka‘opua et al., 2011, 2016; Reading & Wien, 2009; Sotero, 2006). In Hawai‘i, declines in Native Hawaiian health have been attributed to epidemics of foreign diseases, cultural dispossession, land dispossession, and systematic suppression of Native Hawaiian power (Ka‘opua et al., 2017; Riley et al., 2022). Related to these factors, homelessness, which Native Hawaiian youth disproportionately experience, puts youth at exceptionally high risk for sexual assault, survival sex which may result in sexually transmitted infections (STIs) and pregnancies, and challenges accessing sexual and reproductive health (SRH) services (Aparicio et al., 2021; Boutrin & Williams, 2021; CDC, 2021). The current study explores how culturally based approaches enhance SRH programs for Native Hawaiian and Pacific Islander youth. To frame this study, we first explore the relevance of historical and present events concerning the introduction of STIs and health disparities in the Hawaiian islands, the relationships between land dispossession and SRH disparities, the current homelessness crisis among Native Hawaiian and Pacific Islanders in Hawai ‘i, and resistance against cultural and land dispossession.

### Introduction of Foreign Diseases and Sexual and Reproductive Health Disparities to the Hawaiian Islands

Native Hawaiians have grappled with SRH problems and other health consequences of Western colonization since 1778 when British sailors arrived and introduced STIs (Archer, 2018; Ka‘opua et al., 2011). Contact with merchants and missionaries brought additional infectious disease epidemics (Archer, 2018; Ka‘opua et al., 2011). Lack of medical resources and untreated STIs increased infertility, congenital STI transmission, and infant mortality, decreasing the Native Hawaiian population by 90% by 1860 (Archer, 2018; Swanson, 2019). Due to these losses, many Native Hawaiians perceive pregnancy and children as blessings, as they are seen as a connection to ancestors and extensions of family lines (Soon et al., 2015).

### Relationship Between Cultural and Land Dispossession and Sexual and Reproductive Health Disparities

The decline of the Native Hawaiian population was the catalyst for Westerner-led cultural and land dispossession and the subsequent homelessness crisis (Archer, 2018). Cultural dispossession is the loss of cultural roles, language, or religion, while land dispossession refers to land being taken away from Indigenous peoples to establish settler colonial societies (Nichols, 2020; Sotero, 2006). Since some diseases did not spread as quickly among Westerners due to immunity from previous exposure, the Hawaiian royal family sought the advice of Western missionaries and physicians, who blamed the public health crisis on Native Hawaiian culture (Archer, 2018). Legislation was passed that banned public performances of the *hula*, an ancient cultural practice that includes dance accompanied by chant or song (Silva, 2000), and the use of the Hawaiian language in public schools (Goodyear-Kaʻōpua, 2013; Ka‘opua et al., 2016; Reading & Wien, 2009; Sotero, 2006; Trask, 2000).

Denigrating native knowledge and banning cultural practices is a form of cultural dispossession that allows colonizers to readily pursue and justify the dispossession of native lands (Ka‘opua et al., 2016). Cultural dispossession is considered a form of cultural trauma with longstanding effects on present-day health disparities experienced by Native Hawaiians through exposure to stress and stigma and deprivation of cultural and health-related resources (Cook et al., 2003; Subica & Link, 2022).

As more Westerners immigrated and imposed their social and cultural norms, more legislative measures to take land were passed. The Great Māhele separated *makaʻāinana* (people who tend the land, common people) from *ʻāina* (the land) and granted non-Native Hawaiian immigrants and corporations land ownership (Ka‘opua et al., 2011). Native Hawaiian control over lands was further eroded after the illegal, forced overthrow of the Hawaiian Kingdom and annexation to the USA as a territory (Trask, 2000). This history of the USA taking land away from Native Hawaiians contributes to their present-day homelessness crisis (Jensen & Lopez-Carmen, 2022; Koh, 2018).

### Current Homelessness Crisis and Sexual and Reproductive Health Disparities in Hawaiʻi

Homelessness has been declared a state-wide emergency in Hawaiʻi with residents of Native Hawaiian or Pacific Islander descent far more likely to experience homelessness (Perez & Philip, 2020; State of Hawaii, 2022; Yuan et al., 2018). This disparity is also seen across age groups, as a survey conducted by Waikiki Health of youth experiencing homelessness (YEH) who used their services (*N* = 151) from July to October 2016 found that nearly half (44%) identified as being Hawaiian/Part Hawaiian descent (Yuan et al., 2018). The rate is similar to 52% identifying as wholly or partly Native Hawaiian or Pacific Islander in an annual point-in-time (PIT) count of people experiencing homelessness conducted in O‘ahu (Partners In Care & O‘ahu Continuum of Care, 2022). Such high rates are of concern, considering that Native Hawaiian and Pacific Islanders represent 10% of the population in Hawaiʻi (Partners In Care, 2022). Native Hawaiian and Pacific Islanders are over two and a half times more likely to represent individuals in the PIT count than the general population of O‘ahu (Partners In Care & O‘ahu Continuum of Care, 2022).

National-level data suggest that homelessness correlates with young parenthood (Morton et al., 2018), and various studies tell a similar story. A longitudinal study of YEH in the Midwest that collected data from 1999 to 2003 found that 70% of female YEH who are sexually active became pregnant during adolescence (Crawford et al., 2011). A 2016 survey found that in Hawai‘i, 29% of YEH living in O‘ahu had experienced pregnancy (Yuan et al., 2018). Native Hawaiians report the highest unintended pregnancy rate in Hawai‘i, and this disparity can be attributed to financial and social inequities (Office of Hawaiian Affairs, 2018). There is an understanding among Native Hawaiians that pregnancies may not occur under ideal conditions, and since children are seen as blessings, there is an expectation for youth to continue unplanned pregnancies (Soon et al., 2015).

Though STI rates among this population are difficult to obtain, the same survey found that 14% of YEH reported a diagnosis of HIV/AIDS or a physical or developmental disability (Yuan et al., 2018). Despite more Native Hawaiian middle and high school students engaging in conversation with their doctors about STI prevention and with their parents about sex than their non-Hawaiian counterparts, Native Hawaiian middle and high school student reported lower rates of condom use during the last time they had sexual intercourse than their non-Hawaiian peers (Office of Hawaiian Affairs, 2018). Native Hawaiians and other Pacific Islanders report a higher prevalence of HIV (25.3 vs. 9.6 per 100,000 for males and 2.1 vs. 1.7 cases per 100,000 for females in 2018) and higher rates of chlamydia (3.3 times), gonorrhea (2.5 times in males, 2.6 times in females), and primary and secondary syphilis (2.8 times in males, 1.9 in females) than their White counterparts (CDC, 2021). A literature review of studies on STI rates among YEH found rates to range from 6 to 32%, ranging from 17 to 46% among female YEH and 9 to 13% among male YEH (Caccamo et al., 2017).

Risk behaviors for STIs are associated with unmet survival needs and duration of homelessness (Caccamo et al., 2017). Among YEH, there is a high risk of sexual violence and exploitation (Caccamo et al., 2017). In the 2016 survey of YEH living in O‘ahu, 13% reported engaging in survival sex, of which about two-thirds (65%) reported being forced into survival sex (Yuan et al., 2018). Thus, supporting SRH is particularly important for this population.

### Resistance Against Cultural and Land Dispossession

Native Hawaiians have persistently resisted cultural and land dispossession (Pacheco, 2005). This history of resistance is paralleled by increased efforts to integrate Hawaiian cultural values, beliefs, traditions, and consciousness of histories of oppression into state and city-run social services and public health programs for Native Hawaiians (Browne et al., 2017; Mokuau et al., 2012). Examples include Kupu, an environmental and cultural stewardship program for young Native Hawaiian adults (Kupu Hawaii, 2022), and many other culturally based programs addressing various community issues (Chung-Do et al., 2019). The most notable example is the integration of cultural practices into courses offered in jails and prisons housing Native Hawaiians, which began in the early 2000s (Chock, 2022; Ka‘opua et al., 2012). Native Hawaiians are also disproportionately affected by carceral systems (Ka‘opua et al., 2012). Culture-based programs have been found to prevent and control chronic diseases (Browne et al., 2017; Dickerson et al., 2012; Mokuau et al., 2012). These programs highlight how integrating cultural sensitivity to the historical and contemporary marginalization of Indigenous populations can strengthen engagement and reduce health inequities (Jensen & Lopez-Carmen, 2022; Lines et al., 2019; Mokuau et al., 2016).

### Current Study

In light of this broader movement of integrating culture into state and city-run programs, our team saw the potential for embedding cultural practices into Wahine (“woman”) Talk, a multilevel, comprehensive SRH program for female youth experiencing or at risk of homelessness (Aparicio et al., 2018). Wahine Talk was developed to be implemented in any context serving YEH regardless of location and youths’ cultural context. While no female YEH participant indicated disliking the original program, we conducted this study to explore potential ways to enhance cultural attunement in Wahine Talk and help inform SRH programs like it. The primary research question of this study was: *What cultural approaches would Wahine Talk youth participants and program providers like to see included in SRH intervention approaches for Native Hawaiian and Pacific Islander YEH?* This study thus explores the potential for integrating culturally based practices into SRH programs, grounded in the perspectives of youth and SRH program providers living in Hawai‘i.

## Methods

The data for this study are drawn from a mixed-method feasibility study of Wahine Talk, which was created in a university-community partnership to develop, implement, and test the program with and for female YEH. Wahine Talk was iteratively developed and tested from 2016 to 2019 through a community-engaged research-practice partnership between Waikiki Health’s YO! Program and a research team at the University of Hawai‘i, and later, the University of Maryland, College Park.

### Wahine Talk Intervention

Wahine Talk is based on the social-ecological model of behavior change, which posits that factors at various levels, including interpersonal, organizational, community, and public policy levels, support and maintain health-related behaviors and that changes at these levels will produce changes in the individual (McLeroy et al., 1988). Wahine Talk enhances female YEH’s health through four complementary components tailored to each youth’s needs: basic needs services (individual level), SRH education group sessions and peer mentoring (interpersonal level), and linkage to and provision of youth-friendly, trauma-informed SRH care (systems-level) (Aparicio et al., 2018). Wahine Talk’s multi-component approach functions at each level of the socio-ecological model to improve homeless female youths’ overall well-being, linkage to healthcare, and uptake of contraception (Aparicio et al., 2018, 2020; Kachingwe et al., 2022). Data from the current study are drawn from the final year of implementation (2018–2019), wherein the program lasted approximately 3 months and was open to interested female participants of Waikiki Health’s YO! Program between the ages of 14 and 22. Recruitment occurred at the YO! Program’s drop-in center and during community outreach activities.

Wahine Talk youth participants received a free smartphone at enrollment to facilitate program components. The smartphone connected youth with their peer mentor and each other via social media, texts, and calls. Youth also reported using the phone to connect with employers and family members. Program staff provided alternative ways for youth to get these benefits outside of Wahine Talk if youth did not want to participate, such as attending job training or working on-site at the drop-in center (Aparicio et al., 2018, 2020; Kachingwe et al., 2022). The program included group-based SRH educational sessions offered twice weekly, one-on-one mentoring with program staff, and connecting with the peer educator through text and social media. Youth were encouraged to discuss their experiences, engage with guest speakers, and reflect on how they could improve their SRH during group sessions. Participants chose how much they participated in each program component and could receive program incentives if they completed a program checklist where they attended at least four of ten group sessions, had two or more meetings with their peer health educator, met for education and employment counseling once or more, and met with a medical provider once or more. Participants took an average of 12 weeks to complete their program checklists.

### Data Collection

The study team collected qualitative data through ten individual in-depth interviews and three focus group interviews. All year three (2018–2019) youth participants were eligible for an individual in-depth interview and one focus group interview. Ten youths participated in individual and focus group interviews and were not double-counted in our number of participants. Allowing these conversations to happen in individual and group contexts allowed youth to speak up in the format in which they felt most comfortable. The focus group interview allowed the participants to build off one another’s responses. Youth participants received incentives for the individual and focus group interviews. Focus group interviews were held with four providers following program implementation in years two and three. Wahine Talk providers participated in focus group interviews separately from the youth and did not receive incentives for participating.

Interview questions were asked about the Wahine Talk program, the participants’ culture, and how the program could be improved. Participants were asked about their family/community’s culture and beliefs/ideas about sexual health and then asked to compare these ideas to what they had been taught about sexual health by Wahine Talk. Finally, participants were asked if and how they wanted their family/community’s culture to be a larger part of Wahine Talk. Individual interviews lasted an average of 45 min, and focus groups lasted 1 to 2 h.

A research assistant conducted the in-depth interviews, and the focus groups were conducted by the senior author, who led the Wahine Talk evaluation. The research assistant and senior author did not deliver the Wahine Talk intervention. Interviews were held in a private location at the youth drop-in center. Before the start of the focus and individual interviews, interviewers read the consent forms to the participants, answered any questions, and then the participants signed the consent forms.

### Data Analysis

We used an inductive approach and the six steps of thematic analysis described by Braun and Clarke (2006) to analyze the data. The first author initially became familiar with participants’ experiences by listening to audio recordings and reading the transcripts from the ten in-depth and three focus group interviews (Braun & Clarke, 2006). An initial codebook was created and refined after consultation with co-authors (Braun & Clarke, 2006). Next, the first author coded each transcript line-by-line in NVivo, refining the codebook in consultation with the study team to ensure it adequately reflected the participants’ experiences (Braun & Clarke, 2006; QSR International, 2022). Then, the first author collapsed the codes into initial themes (Braun & Clarke, 2006). Afterward, the first author refined the themes, checked them against the extracted codes and the dataset, and developed our final set of themes (Braun & Clarke, 2006). This manuscript represents the final step of reporting results (Braun & Clarke, 2006). The first author regularly brought coding and emergent themes to the study team for review and discussion weekly throughout this process. Thus, themes were developed and refined through collaboration with the study team. The study team kept a detailed audit trail, including the first author’s memos describing the analytic coding process and thematic development and weekly meeting minutes, documenting discussions of coding and emergent themes. The study team engaged in regular peer debriefing and reflexivity throughout the study.

### Positionality Statement

The study team was formed from a research-practice partnership between two universities and a community-based YEH drop-in center managed by a federally qualified health center. The authors include doctoral students, undergraduate students, faculty members, and drop-in center program staff. Our team comprises mainly cisgender women with diverse sexual orientations, racial, ethnic, and cultural backgrounds and identities. Authors have expertise in SRH issues, adolescent health, women’s health, work with Native Hawaiian and Other Pacific Islander youth and families, counseling children and teens who have experienced intra- or extra-familial sexual abuse, and issues affecting YEH. The lead author has a Master of Public Health degree and identifies as bicultural, specifically Taiwanese American, having grown up in the continental United States with parents from Taiwan. None of the authors has experienced homelessness, but all have worked and have expertise with YEH. We have carefully considered our identities and backgrounds during the analysis process, regularly reflecting on our positionality and addressing any biases to help ensure we are authentically representing study participants’ experiences.

## Results

### Sample

The study sample included ten Wahine Talk youth participants and four Wahine Talk program providers. The ten Wahine Talk youth participants were aged 14–22 (*M* = 18.1) years and were a subset of the 17 Wahine Talk youth participants; seven youth did not complete an individual interview or focus group. Although Wahine Talk is not exclusive to Native Hawaiian or Pacific Islanders, all Wahine Talk youth participants in the current study sample were of Native Hawaiian or Pacific Islander ancestry. Some youth participants also had Asian or European ancestry. In addition, six youths had *hānai* (informal adoptive) families (Handy & Pukui, 1953). The Wahine Talk program providers included a peer mentor, a community health educator, a program manager, and a medical provider. The complete demographics of youth participants can be found in Table 1.

**Table 1:** Demographics of youth participants (*n* = 10)

Variable	M (*SD*) or % of sample
Age	18.1 (*2.3*)
Race and ethnicity (categorical)	
Native Hawaiian and other Pacific Islander	70%
Mixed race/ethnicity	30%
Race and ethnicity (all)	
Native Hawaiian	70%
Samoan	30%
Tongan	20%
Micronesian	20%
Chinese	10%
Japanese	30%
Korean	10%
Filipino	10%
White/Caucasian	10%
Sexual orientation	
Bisexual	20%
Heterosexual	80%
Age at first sex	14.1 (*1.8*)
Prior pregnancy	60%
Prior miscarriage	20%
Prior abortion	30%
Prior live birth	40%
Children living with participant	40%
Prior birth control use	20%
Birth family	
Size of birth family	7.8 (*2.1*)
Years of school birth mother completed	11.4 (*2.4*)
Years of school birth father completed	11.8 (*3.3*)
*Hānai* family	
Has a *hānai* family	60%
Age joined *hānai* family	8.7 (*5.4*)
Size of *hānai* family	5 (*1*)
Years of school *hānai* mother completed	11.8 (*.5*)
Years of school *hānai* father completed	12.3 (*.5*)
Adopted family	
Was adopted	20%
Age adopted	1 (*0*)
Size of adopted family	9 (*5*)
Years of school adopted mother completed	12 (*0*)
Years of school adopted father completed	15 (*4.2*)
Foster care	
History of foster care	40%
Age entered foster care	9.5 (*7.2*)

*Hānai* family = informally adopted or chosen family

### Themes

Thematic analysis revealed six themes from youth and providers relevant to integrating culture into SRH programs. One theme, *Estrangement from Culture*, reflects cultural dispossession and highlights the need to incorporate culturally based approaches into SRH. Four themes, *Orientation toward Interdependence*, *ʻĀina-based Approaches*, *Body-Based Approaches*, and *Language*, reflect specific cultural perspectives and practices that can be integrated into SRH programming. Finally, the sixth theme, *Maintaining Boundaries*, describes how cultural practices should be integrated into SRH programming.

Youth suggested including multiple cultural practices that reflect a Native Hawaiian conception of health, *lōkahi*, despite expressing distance from their cultural identity (Martin & Godinet, 2018; Stanford Medicine, 2019). *Lōkahi* is a Native Hawaiian perspective of health that is far more holistic and expansive than a Western biomedical perspective of health (Martin & Godinet, 2018; Stanford Medicine, 2019). These practices enhance YEH’s connection to their bodies, land, and community through several *ʻāina* (“land”)–based approaches, hands-on learning, *hula*, and language. Wahine Talk program providers noted the importance of incorporating culture respectfully, implementing trauma-informed care, and caring for the whole person.

#### Theme One: Estrangement from Culture

Youth participants expressed an estrangement from their ancestral, family, or community cultures. Sometimes, youth explicitly indicated that they did not identify with a particular culture. Other times, the youth specified that they were not raised with ancestral traditions. Several youths expressed that they did not know their ancestral language. For example, one youth demonstrated difficulty identifying a cultural practice and expressed that her family does not maintain tradition. She shared, “No, I don’t think I have [cultural identity] […] [M]y family don’t do any traditions […].” Another youth indicated that she was not raised in ancestral ways and described losing the ability to understand Tongan:

Actually, I-I wouldn’t really know, because, like, we weren’t really raised [in] our Hawaiian or Tongan ways, or Japanese ways. [...] [W]e were just raised like us. No Hawaiian language, no Tongan language. But I know when I was younger, I used to understand Tongan and it just disappeared out of nowhere. I just stopped understanding everything.

#### Theme Two: Orientation Toward Interdependence

*Lōkahi* considers relationships between *kānaka* (people) integral to health (Martin & Godinet, 2018; Stanford Medicine, 2019). Within the concept of *lōkahi*, harmony among *kānaka* promotes health (Martin & Godinet, 2018). Thus, *lōkahi* promotes an orientation toward interdependence, a social norm also found in Pacific Islander cultures (Godinet et al., 2019; Kamaka et al., 2021). Youth participants demonstrated an orientation toward interdependence by asking for content that promotes healthy relationships and asking for more community members to be included as program participants, demonstrating care for community members and recognizing that the well-being of community members matters to their health.

Youth were interested in expanding SRH programs to include content about healthy relationships. When a research team member asked a youth during her interview if she was interested in learning more about healthy relationships based on something the youth mentioned, the youth responded, “[y]eah, I think that would help. With everybody. […] Not just me. ‘Cause [my partner and I have] just stupid little fights. But with everybody else, it's like ‘oh my gosh.’”.

Youth participants also recognized the role of their male counterparts in preventing teen pregnancy when they suggested programming for their male counterparts, “they should have, like, boys’ groups […] [t]hey should really be learning all of this stuff.”

Youth participants demonstrated their care and concern for more community members when they expressed interest in including them in Wahine Talk. Youth participants discussed the need to offer Wahine Talk to community members older than 22 (the current program age limit) because they felt older individuals could also benefit from the program. They shared that “[community members] should come in here. But they’re […] a little older.” This suggestion acknowledges that the SRH-related difficulties of homelessness are not bound by age. Youth participants also emphasized the need to meet people where they were. They suggested doing the work of Wahine Talk at homeless encampments and reaching out to former YEH in foster care who may be unable to go to the drop-in youth center.

Youth participants also thought it was important for couples to have an outlet to express and discuss their feelings about their relationships:

I think like, you know, if a girl is like feeling a certain way towards how they’re being treated [by their partner] [...] and they’re [...] able to come to YO! and explain it to, like, their group leader or whatever, but then like, at the same time, we don’t know if, like, the person that we’re with or our partner is like—is not expressing all their feelings to us, like, if we don’t know how they feel [...] so I think it’s good if [both partners] explain it to the leader, so that we can hear, you know, what’s really how they feel.

The youth demonstrated that they considered relationships and the well-being of community members to be important to them. This orientation toward interdependence was further exemplified by the suggestion that youth participants participate in field trips where they complete outdoor activities like hiking or camping together to “experience [each other’s] strengths and weaknesses.” The youth explained that this would “[bring] a better bond […] ‘cause you’re not only here just talking about something, but you’re, like, pushing each other, like, ‘Oh, let’s go.’”

#### Theme Three: ʻĀina-Based Approaches

Youth frequently recommended *ʻāina*-based approaches. *ʻĀina* refers to land, and *ʻāina*-based approaches acknowledge that people and communities thrive through spending time with *ʻāina* (Maunakea, 2021). *Lōkahi* also emphasizes the importance of relationships between *kānaka* and *ʻāina* to health (Martin & Godinet, 2018; Stanford Medicine, 2019)*. Aloha ʻĀina* refers to deep love and care for the *ʻāina* and is a longstanding Native Hawaiian value and way of being that fosters connection with one another, to the past, and group efforts to honor a reciprocal relationship with *ʻāina*; similar beliefs are found in Pacific Islander cultures (Goodyear-Kaʻōpua, 2013; Ho‘omanawanui, 2008; Ka‘opua et al., 2016; Martin & Godinet, 2018; Pukui, 1983; Pukui et al., 1981).

*ʻĀina*-based approaches identified by the youth included spending time in the *loʻi* (taro patch) and field trips in nature (Knipe, 1989). Spending time in the *loʻi* is particularly reverent from a Native Hawaiian worldview. In a Hawaiian creation story, taro is considered the older sibling of *kānaka* (people), and taro and *kānaka* are responsible for caring for one another (Knipe, 1989). One youth who had experience with *loʻi* encouraged her peers to give *loʻi* a chance, “[l]ike, I’m not one to get dirty, but [the *loʻi* is] funner than you think.” Youth suggested field trips like paddling, camping, and hiking as a group; as one youth shared, “I think we should all go paddling.”

Although some youth had described being estranged from culture, they could identify *ʻāina*-based approaches as a cultural practice, with one participant asking the interviewer, “does exploring count as one? Shoot, I don’t know, like going to, like, different trails?” despite also indicating traditional cultural practices were not common in her family. There was shared excitement when youth who had spent time in Hawaiian immersion schools brought up and described *ʻāina*-based approaches with youth without the same cultural-focused education.

#### Theme Four: Body-Based Approaches

Other cultural approaches that emerged were body-based, including *hula* and hands-on learning, often used in Hawaiian language immersion schools (Edwards, 2016). A youth participant shared *hula* as a cultural practice she identified with and indicated she is interested in her son learning traditional dance, “does *hula* count? [Laughs] Yeah I used to do that, and then I wanted my son to do, you know those guys that do that dance […]? Yeah, I wanted him to do that, but I don’t know.” *Hula* provides an opportunity for movement and a way for youth to integrate Native Hawaiian cultural identity into their bodies.

A youth who attended Hawaiian immersion schools suggested more hands-on approaches in Wahine Talk, “ Maybe more hands-on stuff. […] I love hands-on stuff.’Cause [at] my Hawaiian school, like, most of all our work was, like, hands-on and so, like, people are working on stuff.” The Hawai‘i State Department of Education prioritizes a concept called *ma ka hana ka ʻike* (in doing one learns), where students learn through hands-on activities (Hawaii State Department of Education, 2022). Although youth did not specify how hands-on approaches could enhance SRH programming, one hands-on approach would be to practice communicating about sexual boundaries with partners (Kachingwe et al., 2022).

#### Theme Five: Language

Providers and youth participants suggested incorporating local terms and the Hawaiian language into Wahine Talk. Providers mentioned building rapport through local words and phrases: “So, when you use pidgin, when you use just Hawai[ian] terms, it’s like, ‘Oh, okay, okay. I got it now,’ you know?” Youth participants expressed interest in learning or brushing up on ancestral languages like Hawaiian:

Because you don’t really see that [many] people speaking fluent Hawaiian, you know? That’s, like, our culture, so. And it’s, like, fading away slowly by slowly. [...] I need [to] [...] [g]et my culture back. But sometimes I talk to my daughter in Hawaiian, but, like, I forget, too [...]. So, yeah. I just gotta work on that.

The youth also mentioned that interpretation services would be helpful. For example, a youth from Micronesia mentioned that other youth might need interpretation services, “having an interpreter […] for the ones that doesn’t understand. […] ‘Cause there’s still some that comes here and they don't really speak that good much of English.” Interpretation services could help youth to better and fully access SRH services. These language-based approaches could address cultural dispossession by helping youth feel more connected to their ancestral languages (Ka‘opua et al., 2016).

#### Theme Six: Maintaining Boundaries

Youth and provider participants emphasized that care should be taken to maintain boundaries when incorporating cultural approaches. Youth described boundaries for how content should be delivered and when certain community members should be present. Youth included a boundary between talking and learning about SRH during some culturally based activities they proposed, sharing, “[b]ut then we should have to not talk about sexual life on our hikes and in the water.” Youth also noted that although they were interested in including their children in Wahine Talk, there were some activities for which they would prefer that childcare be provided, “[…] at the same time, they should have daycare for, you know, the parents.”

Providers identified boundaries regarding who should be incorporating culturally based approaches into SRH programs. Providers emphasized that while they wanted to acknowledge and honor culture, they were wary of appropriating culture and open to deferring to another provider with the same cultural background as a youth,

I’m not Hawaiian. And I, you know, I don’t wanna appropriate the culture. And so, it’s like, if it’s someone who is, like, vibing more and would connect more with someone who has that culture and who can really be attuned to it, […] I’ve got, like, this whole staff of people who are just, like, there for support.

## Discussion

Mirroring prior literature, study findings support the importance and need for integrating cultural practices into an SRH program for Native Hawaiian and Pacific Islander YEH (Cook et al., 2003; Subica & Link, 2022). Despite articulating feelings of estrangement from culture, the youth emphasized Native Hawaiian perspectives regarding health. In addition, they reclaimed cultural practices stigmatized by American missionaries and their descendants who annexed Hawai‘i. Finally, the youth named cultural practices that directly challenge the historical land dispossession and imperialism that Native Hawaiians and other Pacific Islanders historically have and continue to face.

*Lōkahi*, a Native Hawaiian health perspective, reflects an understanding that a person is healthy when their physical, mental, and spiritual selves are harmonious (Browne et al., 2017; Martin & Godinet, 2018; Stanford Medicine, 2019). *Lōkahi* emphasizes the importance of relationships between *akua* (God[s]), *kānaka*, and *ʻāina* to health (Martin & Godinet, 2018; Stanford Medicine, 2019). This holistic understanding of health also is part of other Pacific Islander cultures (Godinet et al., 2019).

Difficulty identifying cultural practices or indicating that ancestral ways have not been maintained suggests that the participants or their ancestors have experienced cultural dispossession (Sotero, 2006). Despite evidence of cultural dispossession, the youth participants expressed an understanding of *lōkahi* (balance) during the interviews (Martin & Godinet, 2018; Stanford Medicine, 2019). As *lōkahi* conceptualizes family, community, spiritual realms, and land as interconnected, cultural dispossession can disrupt health (Mokuau et al., 2016). Cultural dispossession is understood to manifest in a plethora of health disparities experienced by Native Hawaiians, higher levels of chronic diseases, substance use disorder, and decreased life expectancy (Mokuau et al., 2016). Other Indigenous populations connected cultural dispossession and negative outcomes related to identity, social inclusion, and wellness (Jensen & Lopez-Carmen, 2022; Lewis et al., 2022).

With the theme of interdependence, the youth asked for content on how to improve their relationships and for programming to include more members of their community. The desire for content for improving relationships with partners reflects *lōkahi*. Youth recommendations to meet people where they are, reach out to former YEH in foster care, and include their male counterparts and children demonstrate the youth’s interest in enhancing the inclusivity and accessibility of Wahine Talk.

The participants advocated for approaches aligned with *Aloha ʻĀina*, a longstanding Native Hawaiian value, and way of being that involves deep love and care for the *ʻāina* through the *ʻāina*-based approaches they suggested (Goodyear-Kaʻōpua, 2013; Ho‘omanawanui, 2008; Ka‘opua et al., 2016; Pukui, 1983; Pukui et al., 1981). The youth recognized the importance of *ʻāina* and *kai* (water) to their well-being and suggested group activities with *ʻāina* and *kai*. The importance of land to Native Hawaiian and Pacific Islander youth is particularly poignant in light of imperial activity from the USA in the Pacific region and considering that many Native Hawaiian and Pacific Islander youth experience homelessness in Hawai‘i. *ʻĀina*-based approaches can help YEH resist the social exclusion which often comes with homelessness and remind them that they still belong in Hawaiʻi and the Pacific region despite homelessness, imperialism, and cultural estrangement. The relationship between *ʻāina* and health has been supported by prior research (Antonio et al., 2020; Keli‘iholokai et al., 2020). Other Indigenous youths have also identified a connection to the land as related to health. Specifically, Yellowknives Dene First Nation youth have identified connection to the land as their most important social determinant of health (Lines et al., 2019).

The *ʻāina*-based approaches build upon the orientation toward interdependence theme as these activities are meant to be completed together. Some activities, like spending time in the *loʻi*, emphasize interdependence between *ʻāina* and *kānaka*. In the themes regarding orientation toward interdependence and *ʻāina*-based approaches, youth demonstrated communal and interdependent perspectives of health that differ from a traditional Western biomedical model that tends to be more individualized (Kitayama et al., 2010). The theme of interdependence aligns with the work of a 2015 consensus panel of Indigenous scholars and allied settler individuals who examined the appropriateness of the World Health Organization’s Social Determinants of Health framework for Indigenous populations. The panel declared interdependence an important determinant of health shared by many Indigenous communities in the USA (Carroll et al., 2022).

The body-based approaches and need for linguistic competency identified by youth were stigmatized and formerly banned due to Western influences. However, reclaiming these practices can enhance youths’ relationships with their ancestors and a sense of belonging and help them resist cultural dispossession (Ka‘opua et al., 2016). Furthermore, this suggested approach aligns with other research which has found reclamation of ancestral practices to promote resilience and address health inequities among Indigenous communities (Keli‘iholokai et al., 2020).

Youth and providers emphasized boundaries regarding what content should be delivered together, when certain community members should participate, and who should provide culturally based approaches. These recommendations highlight how culturally based approaches must be thoughtfully integrated into SRH programs and align with calls for cultural safety (Ka‘opua et al., 2017). In Aotearoa (New Zealand), the term cultural safety, known as *kawa whakaruruhau*, emerged when Māori nurses observed their non-Māori colleagues blame poor health outcomes on the culture of their Indigenous patients (Ka‘opua et al., 2017; Papps & Ramsden, 1996). Culturally safe interventions subvert unequal power relationships and honor a group’s strengths in surviving cultural trauma and present-day marginalization (Ka‘opua et al., 2017). Ultimately, when providers are of different cultural orientations from youth, it is up to the youth to define safety (Ka‘opua et al., 2017), and providers should seek their input (Goodyear-Kaʻōpua, 2013; Ho‘omanawanui, 2008; Ka‘opua et al., 2017).

### Strengths and Limitations

The findings from this study should be understood in the specific context in which it was conducted. This study supports a small but growing field of research addressing contemporary health inequities caused by historic and ongoing cultural and land dispossession and approaches all aspects of research, policy, and practice with an awareness of how Western research methodologies are used to silence Indigenous knowledge and cultural practices. This study also expands the knowledge base of health promotion practices specific to Pacific Islander and Native Hawaiian health. Wahine Talk youth participants interviewed for this study were young Native Hawaiian or Pacific Islander women and girls experiencing homelessness in and around Honolulu, Hawai ‘i. The youth participants’ specific contexts and experiences informed these findings, and our study can be instrumental in better understanding and developing interventions for this specific population. For instance, the finding of estrangement from culture may not show up for Native Hawaiian and Pacific Islander youth in higher resource settings. Furthermore, the youth in this study may not reflect all YEH in Hawaiʻi; it is always possible that other youth would express different opinions. Finally, this study examines cultural approaches to SRH programming. Efforts were made to uplift the voices of marginalized Native Hawaiian and Pacific Islander youth; however, most of the study team lacks a Native Hawaiian or Pacific Islander background. We note that because of the Western influence on research institutions, even as we attempt to uplift the voices of this population, we may not understand the whole cultural context as “outsiders.” Our interview guide may not have covered all relevant elements of culture, or the youth may have chosen not to mention certain aspects of their experience that would have been relevant.

### Implications

To adequately address estrangement from culture that the youth describe through the other themes (orientation toward interdependence, *ʻāina*-based approaches, body-based approaches, language, and maintaining boundaries), there are a number of practice, research, and policy implications that we elucidate below. Addressing these themes can improve SRH of Native Hawaiian and Other Pacific Islander youth.

#### Practice Implications

Practitioners must understand the historical and current-day forces that create distance from culture and practice cultural safety and, therefore, not pathologize culture as being the cause of health disparities (Ka‘opua et al., 2017; Knibb-Lamouche, 2013; Subica & Link, 2022). Instead, providers should make services welcoming by respecting all ways of knowing, being open to learning from clients, and monitoring their negative biases against marginalized people (Ka‘opua et al., 2017). Providers can demonstrate respect for all ways of knowing and their openness to learning from clients by practicing proper social etiquette and learning about community history and lifeways (Ka‘opua et al., 2017). For example, Native Hawaiians living in Hawaiian homestead communities suggested that cultural safety includes respectful conversation and suspending research activities on Sundays when families typically gather (Ka‘opua et al., 2017). Monitoring negative bias involves interrogating any biased beliefs providers might hold. Those working with YEH can participate in culturally grounded processes to subvert unequal power relationships, develop trust, and navigate disagreement.

When designing interventions for cisgender female Native Hawaiian or Pacific Islander YEH such as those youth in our study, practitioners should consider adopting a more holistic understanding of health that acknowledges the interdependence of peoples and promotes *‘ike ʻāina* (knowledge from/about land) (Ho‘omanawanui, 2008). *‘Ike ʻāina* supports ancestral knowledge, self-esteem, and well-being among Native Hawaiian students (Ho‘omanawanui, 2008). *‘Ike ʻāina* can involve learning about cloud formations, ocean currents and tides, the Hawaiian language, and *hula* (Ho‘omanawanui, 2008).

There is great potential for incorporating cultural approaches within these programs when completed in a culturally attuned and respectful way, with substantial involvement from those within the community. Many of the practice implications mentioned in this section align with all the principles of trauma-informed care, which include Trustworthiness and Transparency; Peer Support; and Cultural, Historical, and Gender Issues (Substance Abuse and Mental Health Services Administration, 2014). Expanding practice with these suggestions can address SRH among Native Hawaiian and Other Pacific Islander youth by increasing accessibility and acceptability of SRH services and by including male partners in efforts to prevent unintended pregnancy.

#### Policy Implications

Policymakers should address land dispossession to decrease homelessness and promote health through *ʻāina*-based activities (County Health Rankings, 2022; Harvard Law Review, 2020). Unlike some Native American tribes, the US government has not formally recognized Native Hawaiians nor extended land rights and sovereignty (County Health Rankings, 2022). Addressing land dispossession of Native Hawaiians through substantive land reparations could improve the economic conditions of Native Hawaiians as it has done so for other Indigenous populations (Chitonge, 2022; County Health Rankings, 2022; Harvard Law Review, 2020).

Policymakers should also consider integrating cultural practices and substantive presentations of Hawaiian history into public education. A recent evaluation of a cultural and language education program for Cherokee youth found improved diet and exercise, mental health, and social and cultural connection (Lewis et al., 2022). Programs supporting Indigenous language maintenance and revitalization have also been found to protect indigenous health on both an individual and community-wide level (Whalen et al., 2022). Policies expanding and funding existing Hawaiian language immersion programs for youth could provide a similar opportunity for increasing cultural connection, peer support, and community-building. The elimination of policies that disrupt and disband communities, including the City of Honolulu’s police-enforced sweeps of encampments of adults and youth experiencing homelessness, could also foster stronger connections between youth and elders, and sharing cultural knowledge and practices (Perez & Philip, 2020).

Recently, the Hawaiian State Legislature approved a bill for a prison rehabilitation program for Native Hawaiians in 2022, emphasizing Native Hawaiian cultural practices and values (Chock, 2022; Hawai‘i State Legislature, 2022). Policymakers could similarly call for and set aside funds that emphasize cultural practices and values in SRH programs relevant to the local communities being served. In addition, policymakers could set aside funds to develop, evaluate, and disseminate such programs. These policy suggestions can address SRH by stabilizing housing and increasing knowledge of and access to Native Hawaiian ways of understanding and promoting SRH.

#### Research Implications

This research demonstrates that youth participants value and believe culturally based approaches should be integrated into SRH programs despite reporting feelings of estrangement from culture. This study shows that input from community insiders is integral to developing these culturally relevant interventions and services to prevent the perpetuation of cultural stereotypes or cultural appropriation. Our study emphasizes the need for youth voices to create these programs that fit their circumstances and needs. In a research setting, issues will arise if youth feel uncomfortable sharing their experiences and leave out parts of their experiences. Therefore, researchers need to build and maintain a professional and approachable environment for candid discussions. This study only recruited cisgender girls, so the perspectives and needs of male, transgender, and non-binary youth need further evaluation are left out and should be explored in future studies, particularly since expanding Wahine Talk to include more community members was important to youth participants. Also, although some girls identified as bisexual or queer in this study, studies that specifically recruit lesbian, gay, and bisexual youth will be needed to create SRH programs for sexual minority youth. Future research can also explore the intersections between Native Hawaiian and Pacific Islander identity and other aspects of YEH identity that impact SRH.

Themes from our study may be helpful in the development of SRH programming for youth in other contexts. Though the specific themes of this study are relevant to Native Hawaiian and Pacific Islander culture, the results may resonate with other youth from Indigenous communities that have also experienced similar cultural and land dispossession, particularly the themes related to land and interdependence and health promotion (Greenwood & Lindsay, 2019; Knibb-Lamouche, 2013). Further research should provide more directions for improving SRH among Native Hawaiian and Other Pacific Islander youth.

## Conclusion

Despite reporting estrangement from ancestral culture, Wahine Talk youth participants articulated an understanding of Native Hawaiian and Pacific Islander conceptions of health and demonstrated resistance to cultural dispossession. Indigenous Hawaiian and Pacific Islander perspectives recognize *ʻāina* and relationships as connected to SRH. Land sovereignty and inequitable rates of homelessness remain ongoing legacies of colonization in Hawai‘i, impacting SRH among YEH.
