# Correction: “I Need to Get My Culture Back”: Youth and Provider Perspectives on Integrating Culturally Based Approaches into Sexual and Reproductive Health Programs for Native Hawaiian and Pacific Islander Youth Experiencing Homelessness

**DOI:** 10.1007/s11121-023-01630-1

**Published:** 2023-12-22

**Authors:** Charlene Kuo, Michelle Jasczynski, Jee Hun Yoo, Jennifer L. Robinson, Katelyn Reynolds, Lisa Anoruo, Kayla Bae, Lana Sue Ka’opua, Rebecca Chavez, Jacqueline Tellei, Elizabeth M. Aparicio

**Affiliations:** 1 School of Public Health, Department of Behavioral and Community Health, University of Maryland, College Park, MD USA; 2 College of Computer, Mathematical & Natural Sciences, Department of Biology, University of Maryland, College Park, MD USA; 3 School of Public Health, Department of Public Health Science, University of Maryland, College Park, MD USA; 4 Thompson School of Social Work & Public Health (Retired), Department of Social Work, University of Hawai‘i at Mānoa, Honolulu, HI USA; 5 Waikiki Health, Honolulu, HI USA; 6 PATH Clinic and Youth Outreach, Honolulu, HI USA

**Correction to: Prevention Science** 10.1007/s11121-023-01573-7

The article ““I Need to Get My Culture Back”: Youth and Provider Perspectives on Integrating Culturally Based Approaches into Sexual and Reproductive Health Programs for Native Hawaiian and Pacific Islander Youth Experiencing Homelessness”, written by Kuo, C., Jasczynski, M., Yoo, J.H., Robinson, J.L., Reynolds, K., Anoruo, L., Bae, K., Ka’opua, L.S., Chavez, R., Tellei, J., and Aparicio, E.M., was originally published electronically on the publisher’s internet portal on 11 August 2023 without open access. With the author(s)’ decision to opt for Open Choice the copyright of the article changed on 06 December 2023 to © The Author(s) 2023 and the article is forthwith distributed under a Creative Commons Attribution 4.0 International License, which permits use, sharing, adaptation, distribution and reproduction in any medium or format, as long as you give appropriate credit to the original author(s) and the source, provide a link to the Creative Commons licence, and indicate if changes were made. The images or other third party material in this article are included in the article’s Creative Commons licence, unless indicated otherwise in a credit line to the material. If material is not included in the article’s Creative Commons licence and your intended use is not permitted by statutory regulation or exceeds the permitted use, you will need to obtain permission directly from the copyright holder. To view a copy of this licence, visit http://creativecommons.org/licenses/by/4.0/.

The original article has been corrected.

